# Investigating the value of radiomics stemming from DSC quantitative biomarkers in IDH mutation prediction in gliomas

**DOI:** 10.3389/fneur.2023.1249452

**Published:** 2023-11-16

**Authors:** Georgios S. Ioannidis, Laura Elin Pigott, Michael Iv, Katarina Surlan-Popovic, Max Wintermark, Sotirios Bisdas, Kostas Marias

**Affiliations:** ^1^Computational BioMedicine Laboratory (CBML), Institute of Computer Science, Foundation for Research and Technology—Hellas (FORTH), Heraklion, Greece; ^2^Institute of Health and Social Care, London South Bank University, London, United Kingdom; ^3^Faculty of Brain Science, Queen Square Institute of Neurology, University College London, London, United Kingdom; ^4^Lysholm Department of Neuroradiology, The National Hospital for Neurology and Neurosurgery University College London, London, United Kingdom; ^5^Department of Radiology, Division of Neuroimaging and Neurointervention, Stanford University, Stanford, CA, United States; ^6^Department of Radiology, Faculty of Medicine, University of Ljubljana, Ljubljana, Slovenia; ^7^Department of Neuroradiology, University Medical Centre, Ljubljana, Slovenia; ^8^Department of Brain Repair and Rehabilitation, Queen Square Institute of Neurology, UCL, London, United Kingdom; ^9^Department of Neuroradiology, The National Hospital for Neurology and Neurosurgery, University College London NHS Foundation Trust, London, United Kingdom; ^10^Department of Electrical and Computer Engineering, Hellenic Mediterranean University, Heraklion, Greece

**Keywords:** dynamic susceptibility contrast MRI, gliomas, radiogenomics, IDH mutation, generalizability, explainability

## Abstract

**Objective:**

This study aims to assess the value of biomarker based radiomics to predict IDH mutation in gliomas. The patient cohort consists of 160 patients histopathologicaly proven of primary glioma (WHO grades 2–4) from 3 different centers.

**Methods:**

To quantify the DSC perfusion signal two different mathematical modeling methods were used (Gamma fitting, leakage correction algorithms) considering the assumptions about the compartments contributing in the blood flow between the extra- and intra vascular space.

**Results:**

The Mean slope of increase (MSI) and the K_1_ parameter of the bidirectional exchange model exhibited the highest performance with (ACC 74.3% AUROC 74.2%) and (ACC 75% AUROC 70.5%) respectively.

**Conclusion:**

The proposed framework on DSC-MRI radiogenomics in gliomas has the potential of becoming a reliable diagnostic support tool exploiting the mathematical modeling of the DSC signal to characterize IDH mutation status through a more reproducible and standardized signal analysis scheme for facilitating clinical translation.

## 1 Introduction

Gliomas are the most frequent type of brain tumors, which are classified with a grading system outlined by the WHO, ranging from Grade I to IV (1). Diagnosis and classification of gliomas have, as of 2016, incorporated molecular markers, due to the correlation between tumor biology and behavior (2). One of the most important genetic markers seen in gliomas is the isocitrate dehydrogenase (IDH) mutation, which has long seen better patient prognosis than patients with gliomas of an IDH wildtype (3, 4). This mutation is suspected to result in decreased NADPH production within the cell, thus exposing the tumors cells to damage caused by reactive oxygen species (ROS). Whereas, IDH wild-type tumors are more resistant to ROS, likely due to the protection provided by NADPH, and therefore present in a more aggressive form (5).

The identification of IDH mutations is therefore instrumental in predicting patient prognosis, glioma surveillance (2), and potentially treating cancer (6). However, the process of distinguishing IDH mutations consists of invasive biopsies, which are accompanied by limitations such as postoperative complications and clerical or histological errors (7). Recent AI advancement in imaging studies have given rise to a less invasive process to identify potential molecular markers. This is proposed by correlating imaging features, such as, radiological enhancement of the tumor and calcification, with suspected biomarkers, such as an IDH mutation (8, 9). However, although this mechanism of biomarker identification is promising, it may be inconsistent. To this end, there is a need for advancements in machine learning and radiomics in order to provide a more robust and systematic approach to identifying molecular markers and therefore predicting survival outcomes and patient prognosis.

Dynamic Susceptibility Contrast (DSC) perfusion imaging, specifically rCBV maps, can identify defined angiogenesis transcriptome signatures, which could be indicative of IDH mutant gliomas through the evident perfusion phenotypes (10). This indicates that identifiable imaging biomarkers might be detectable from the development of pathobiological tumor vasculature (11), in addition to the imaging features previously mentioned, with the use of multiparametric maps from DSC imaging. However, limited evidence exists on the use of DSC perfusion imaging in radiogenomic studies detecting IDH mutations from glioma vasculature.

Artificial Intelligence is quickly becoming a field of research which could potentially inform clinical decision making processes. It uses radiological images as minable databases that utilize quantitative data that can, once learned, predict clinically relevant information (12). Machine Learning (ML) has been shown to enhance the identification of IDH mutation statuses, through extraction of quantitative data from conventional and advanced magnetic resonance imaging (MRI) techniques using multiparametric maps (13–24). Although these studies assessed the value of machine learning using conventional and multiparametric MRI to predict the presence of IDH mutations in gliomas, only a few of them included multicentric data, which is imperative for a standardized approach for implementation in a future healthcare setting. This is an evident limitation in neuro-oncology radiomics studies, since only 3.9% (2/51) of studies were validated with multicentric data (25).

Therefore, and to the best of our knowledge, the use of DSC perfusion imaging with machine learning to predict IDH mutation status has only been investigated in four studies thus far (18, 21, 26, 27). However, only two publications have investigated the sole use of DSC (18, 21). Promising results were reported in these two studies, however, both studies note the heterogeneity of images from different centers as a factor which could decrease sensitivity and specificity.

The heterogeneity in MRI scanner characteristics, such as software variations, MRI equipment (receiver coils), scan protocols, and reconstruction algorithms, can lead to inter- and intra-site variations. These variations affect the signal intensity of the MR image, which may conceal the region of interest in terms of signal to noise ratio (SNR) and lead to the failure of (ML) analysis (28). To account for this heterogeneity, harmonization and normalization techniques are applied to the data as a pre-processing step which is not intuitive since there is variety of normalization choices that can lead to different results. In addition, another limitation is the lack of standardized radiomic pipelines and explainability mechanisms to increase trustworthiness and accelerate clinical adoption.

This radiogenomics study aims to explore the value of using machine learning (ML) directly on features from DSC parametric maps, derived from mathematical signal modeling, to predict IDH mutation status in gliomas. This methodology is applied on a previously studied dataset (21) as a more standardized and reproducible ML/Radiomics scheme without any pre-processing step.

## 2 Materials and methods

### 2.1 Patient population

The patient cohort consisted of 160 patients [age: 58.4 ± 15.9 (mean ± SD), 70 female] from three different imaging centers. Each patient underwent histopathological diagnosis of primary glioma (WHO grades 2–4), molecular characterization of IDH mutation status (IDH-mutant = 41, IDH-wildtype = 119) and DSC–MRI prior to any treatment. The first cohort consisted of 92 patients (66 out of 92 IDH-mutant) from Stanford Medicine Imaging Center, Stanford CA, USA. The second cohort included 50 patients (39 out of 50 IDH-mutant) from Health Lancaster Imaging Center, South Carolina, USA. The third cohort contained 14 out of 18 patients with an IDH-mutant status from Ljubljana University Medical Center, Ljubljana, Slovenia. Patients without a histologically confirmed diagnosis of glioma, incomplete molecular characterization of IDH status, non-enhancing grade III gliomas, or patients having received any treatment prior to image acquisition were excluded.

### 2.2 MRI protocol

The imaging parameters for each DSC acquisition for each cohort are summarized in Table 1.

**Table 1:** MR imaging parameters per cohort used.

**Scanner type**	**First cohort**	**Second cohort B**		**Third cohort C**
**3T discovery MR750 (GE healthcare, United States)**	**3T siemens skyra (siemens healthineers, Erlangen, Germany)**	**1.5T magnetom avanto (siemens healthineers, Erlangen, Germany)**	**1.5.T Philips Achieva (philips medical systems)**
Acquisition type	2D Echo-Planar Imaging (EPI) with fat suppression (FS)			
Magnetic field strength	3T	3T	1.5T	1.5T
Repetition time (ms)	1800	1870	1850	1525
Echo time (ms)	40	30	30	40
Echo train length	1	63	1	47
Flip angle (deg)	60	90	90	75
In-plane resolution (mm^2^)	1.718 × 1.718	1.719 × 1.719	1.796 × 1.796	1.75 × 1.75
Number of averages	1	1	1	1
Image slice thickness (mm)	5	5	5	5
Image slice spacing (mm)	5	5	5	5
Temporal resolution	60 × 1.87 s	60 × 2.07 s	40 × 1.53s	60 × 1.8s
Matrix size	128 × 128	128 × 128	128 × 128	128 × 128

### 2.3 Tumor delineation

Bratumia software (https://www.nitrc.org/projects/bratumia) (28), was used to delineate the regions of interest (ROIs). This software uses as input four different MR contrasts (T1 before and after contrast, T2, T2 FLAIR) in order to correctly identify tumor enhancement, oedema and necrosis. As a next step three expert neuroradiologists visually inspected the produced tumor ROIs and proceeded to corrections when necessary. Finally, the whole tumor ROI was selected as the combination of tumor enhancement, oedema and necrotic regions.

### 2.4 Parametric map computation

#### 2.4.1 Gamma fitting algorithm

DSC MRI and Computed Tomography perfusion (CTP) are the most widely used tracer kinetic techniques to measure brain perfusion. They both examine how the injected contrast agent is distributed and diluted inside the vascular system (29). To quantify the DSC signal we used an in-house software (30) developed in Python 3.5 (www.python.org) mainly for CTP. The quantification software was based on the work of Meier and Ziegler (31) and is briefly described below.

Given a probability density function or transport function *h*(*t*) that describes the transit of CA particles in the vascular system, the equation that relates blood flow (*F*_*t*_) with the concentration of CA in the tissue *C*_*t*_(t) is given by the formula below:


(1) $$\begin{array}{c} C_{t}\left(t\right)=F_{t}\;AIF⊛\text{R}\left(\text{t}\right), \end{array}$$

where, *AIF* is the arterial input function, ⊛ is the convolution operator and $R\left(t\right)=1-\int_{0}^{t}h\left(\tau \right)d\tau$ is the residue function that denotes the amount of CA that is still present in the volume of interest at time *t* (32). To account for dispersion effects (33) the gamma variate function was chosen as the transport function *h*(*t*) (34).


(2) $$\begin{array}{c} h\left(t\right)=\;\{\;\begin{array}{c} \frac{1}{A_{1}}{\left(t-t_{1}\right)}^{a_{1}}\;e^{\frac{-\left(t-t_{1}\right)}{\sigma_{1}}},\;\left(t\geq t_{1}\right)\;\\ 0,\;\left(t<t_{1}\right)\; \end{array} \end{array}$$

where, $A_{1}\;=\sigma_{1}^{1+a_{1}}\;\Gamma \left(1+a_{1}\right)$, Γ(*a*) is the Gamma probability density function, *a*_1_, σ_1_ and *t*_1_ are related with the mean transit time and the dispersion of *h*(*t*).

In order to obtain [*F*_*t*_, *t*_1_, σ_1_, *a*_1_], the (scipy.optimize.least_squares) (35) was used to fit Equation 1 to the DSC concentration curves (6) in a voxel by voxel basis. Finally, by applying the central volume principle relative cerebral blood flow (rCBF), blood volume (rCBV) and mean transit time (rMTT) were calculated as


(3) $$\begin{array}{c} rCBF=\;F_{t} \end{array}$$

(4) $$\begin{array}{c} rMTT\;=\;t_{1}+\sigma_{1}\left(1+a_{1}\right) \end{array}$$

(5) $$\begin{array}{c} rCBV=rMTT\;*\;F_{t}\;\left(\text{central volume principle}\right) \end{array}$$

In addition, the TMAX and the mean slope of increase (MSI) were also calculated. TMAX represents the time taken for the DSC curve to reach its maximum. Assuming *C*_*t*_(*t*) to be the perfusion curve and *t*_0_ the last time of the baseline, MSI was calculated as:


(6) $$\begin{array}{c} \;MSI\;=\frac{1}{N}\overset{t_{N}\;=\;TMAX}{\underset{{t_{1}=\;t}_{0}}{\sum }}C_{t}\left({\text{t}}_{\text{i}+1}\right)-C_{t}\left({\text{t}}_{i}\right) \end{array}$$

It is also important to note that, the concentration of CA over time [*C*_*t*_(*t*)] for each voxel in a DSC series [S(t)] is assumed to be proportional to the change of relaxation rate in the tissue as expressed in Equation 1:


(7) $$\begin{array}{c} C_{t}\left(t\right)\;\propto \Delta R_{2}^{*}\left(t\right)=\;-\frac{1}{TE}\;\ln\left(\frac{S\left(t\right)}{S\left(0\right)}\right) \end{array}$$

where TE is the echo time and S(0) is the baseline signal prior to the CA's arrival. Thus, prior to fitting every DSC intensity curve was converted to concentration of CA using Equation 7 (29).

#### 2.4.2 Leakage correction algorithms

To determine the relative cerebral blood volume (nrCBV) is a challenging task in brain tumors since a leaky blood-brain barrier (BBB) can affect measurements (36). That said, lesions with a disrupted BBB, permit CA leakage into the extravascular extracellular space (EES), reducing both T2^*^ time further. Thus, a more complex model must be taken into account to allow the uni- or bi-directional exchange of CA between EES and intravascular space (IVS). Thus, multi compartmental modeling is presented below for the calculation of the corrected nrCBV (37).

In general, nrCBV is the integral of *C*_*t*_(*t*) (Equation 7) between the arrival *t*_0_ and the replenish *t*_1_time points as shown in Equation 8 below:


(8) $$\begin{array}{c} \;nrCBV=\;\int_{t_{0}}^{t_{1}}C_{t}\left(t\right)\;dt \end{array}$$

In the unidirectional leakage correction algorithm only the transfer from IVS to EES is assumed and *C*_*t*_(*t*) is modeled as a linear combination of the whole brain average concentration $\bar{C_{t}\left(t\right)}$ in non-enhancing voxels and its time integral in the next equation:


(9) $$\begin{array}{c} \;C_{t\;}\left(t\right)=K_{1}\;\bar{C_{t}\left(t\right)}-K_{2}\;\int_{0}^{t}\;\bar{C_{t}\left(\tau \right)}d\tau \end{array}$$

where, $K_{1}\;\left(\sec^{-1}\right)$ is a susceptibility scaling factor and $K_{2}\;\left(\sec^{-1}\right)$ is a permeability related parameter for intra- to extravascular contrast flow. *K*_1_ and *K*_2_ are obtained by fitting equation 9 to the concentration *C*_*t*_(*t*) voxels over time inside the region of interest (ROI). Thus, the unidirectional corrected time curve *C*_*t unidir*_(*t*) and *nrCBV*_*unidir*_ can be computed for each voxel from the next two equations:


(10) $$\begin{array}{c} C_{t\;unidir}\left(t\right)=C_{t\;}\left(t\right)+K_{2}\;\int_{0}^{t}\;\bar{C_{t}\left(\tau \right)}d\tau \end{array}$$

(11) $$\begin{array}{c} \;nrCBV_{unidir}=\;\int_{t_{0}}^{t_{1}}C_{t\;unidir}\left(t\right)\;dt \end{array}$$

In the case of the bidirectional corrected algorithm the bidirectional transfer between EES and IVS is assumed and *C*_*t*_(*t*) is modeled by adding an extra term in Equation 9 as follows:


(12) $$\begin{array}{c} C_{t\;}\left(t\right)=K_{1}\;\bar{C_{t}\left(t\right)}-K_{2}\;\bar{C_{t}\left(\tau \right)}{\;⊛\;e}^{-K_{ep}} \end{array}$$

where *K*_*ep*_ is the transfer constant for extra- to intravascular compartments and ⊛ is the convolution operator. Again, *K*_1_, *K*_2_ and *K*_*ep*_ are obtained by fitting Equation 12 to the concentration *C*_*t*_(*t*) voxels over time inside the region of interest (ROI). Thus, the bidirectional corrected time curve *C*_*t bidir*_(*t*) and *nrCBV*_*bidir*_ can be computed for each voxel from the next two equations:


(13) $$\begin{array}{c} C_{t\;bidir}\left(t\right)=\;\bar{C_{t}\left(t\right)}+K_{2}\;\bar{C_{t}\left(\tau \right)}{\;⊛\;e}^{-K_{ep}} \end{array}$$

(14) $$\begin{array}{c} nrCBV_{bidir}=\;\int_{t_{0}}^{t_{1}}C_{t\;bidir}\left(t\right)\;dt \end{array}$$

**Figure 1:**
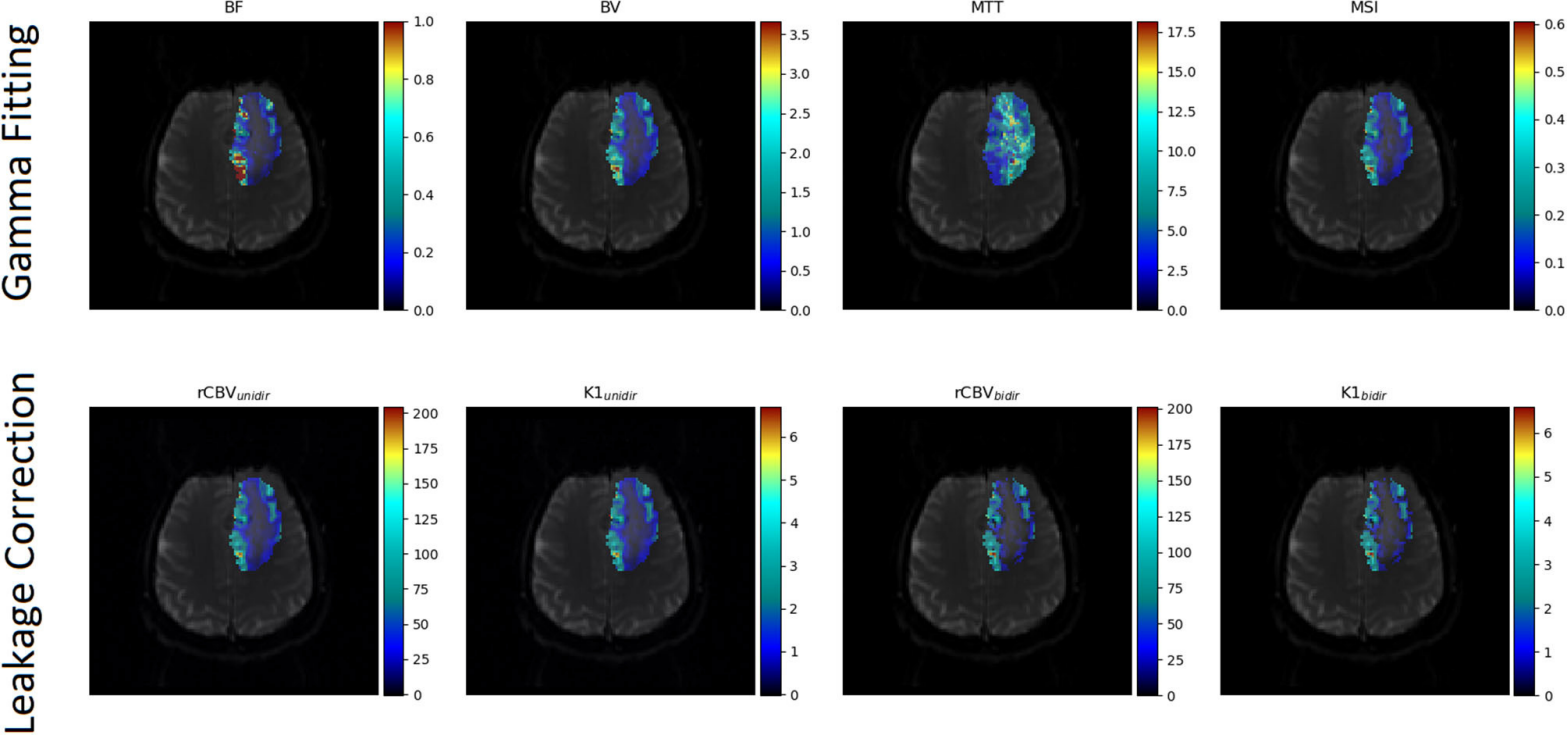
Parametric maps calculated from the gamma fitting and the leakage correction algorithms superimposed to the corresponding anatomical image.

For the leakage corrected algorithms the search space for the unknown fitted parameters *K*_1_, *K*_2_ and *K*_*ep*_ was the real numbers without any constrains with the Levenberg-Marquardt algorithm (38) to account for $T_{2}^{*}$ leakage effects. Some of the aforementioned parametric maps are depicted in Figure 1.

### 2.5 Radiomic features

For each of the aforementioned parametric maps (rCBF, rCBV, rMTT, MSI, TMAX and nrCBV, K1unidir, K2unidir, nrCBVunidir, K1bidir, K2bidir, Kepbidir, nrCBVbidir) described in section 2.4 the pyradiomics library (39) was used to extract features based on the annotations described in section 2.3. All the available features classes from the pyradiomics library were incorporated including: (a) statistical features such as first order statistics and higher order statistics, (b) texture features such as Gray-Level Run Length Matrix (GLRLM), Gray-Level Co-Occurrence Matrix (GLCM), Gray Level Size Zone Matrix (GLSZM) and Gray Level Difference Matrix (GLDM) and (c) shape-based 2D and 3D features. Additionally, local binary patterns 2D (LBP) and image transformation methods such as Laplace of Gaussian (LoG), Logarithmic, Exponential, and Gradient were used leading to 1,734 features.

### 2.6 Feature selection

The feature selection process used in this study consisted of 3 steps. The first step was to apply a variance threshold to remove features with zero variance (constant features with variance lower than 0.5). Secondly, a univariate method (ANOVA, analysis of variance) was used to remove noisy information in a feature by feature basis. The last step was to apply a multivariate method (linear logistic regression) using the l1 norm (l1 penalty) that produces the feature importance weights by solving a minimization problem using as penalty parameter C = 0.3 (40). After that the SelectFromModel function from the sklearn library is used as a meta-transformer for selecting features based on the aforementioned importance weights. Further information about the feature selection method can be found in Trivizakis et al. (41).

### 2.7 Synthetic minority oversampling technique

A common problem in classification analyses is the imbalanced number of samples in each class. In our study, we had 41 IDH-mutant and 119 IDH-wildtype cases which can lead to a biased machine learning classifier with reduced sensitivity. To overcome this limitation, the synthetic minority oversampling technique (SMOTE) was applied on the training phase of the classification and the trained models were evaluated exclusively on the unseen testing sets (42). SMOTE works by selecting samples from the minority class that are close in the feature space. It draws a line between the samples in the feature space and produces a new sample at a point belonging to that line.

### 2.8 IDH classification

**Figure 2:**
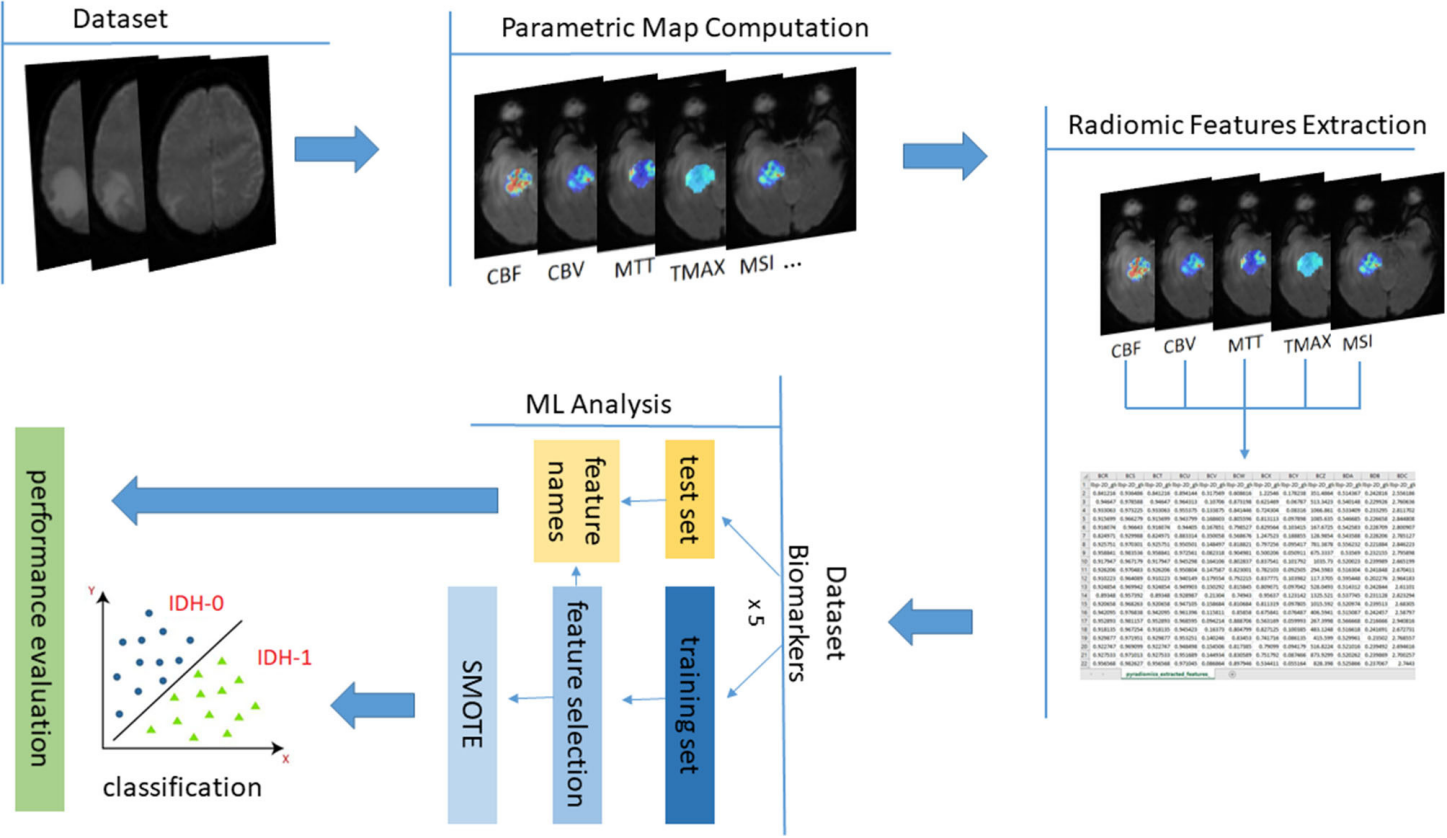
The overall data analysis process with the proposed MRI parametric maps for IDH prediction.

In order to differentiate the IDH mutation status, the support vector machine (SVM) classifier with the radial basis function kernel (RBF) from the scikit-learn library (43) was used. Support vector machines (SVM) have been widely used in medical image classification problems (44–46). The SVM classifier was trained in a 5-fold cross-validation scheme on the imaging biomarker features stemming from the radiomic analysis. To avoid sample selection bias and overfitted models the data stratification was applied on a patient basis with respect to the class representation across folds. The overall workflow is illustrated in Figure 2.

### 2.9 Explainability and model performance evaluation metrics

To evaluate and compare the performance of radiomic analysis in the produced biomarkers with other studies in bibliography a variety of metrics were used. More specifically, for every fold, sensitivity, specificity, F1-score, accuracy (ACC) and area under the receiver operating characteristic curve (AUC) with their standard deviations were calculated on the unseen testing sets. The performance metrics are defined as: sensitivity $=\;\frac{TP}{TP+FN}$, specificity $=\;\frac{TN}{FP+TN}$, F1-score $=\;\frac{TP}{TP+0.5\left(FP+FN\right)}$ where, TP, TN, FP, and FN stand for true-positive, true-negative, false-positive and false-negative respectively. The ROC curve is a two-dimensional graph in which the y-axis indicates the true positive rate and the x-axis the false positive rate and the AUC has been extensively used to evaluate MLframeworks.

To assess the explainability of the model, the SHAP method (Shapley Additive Explanations) was used to explain individual predictions. SHAP values are not model dependent, meaning they can be used to interpret any machine learning model. Their background relies on a game theoretic approach that measures the contribution of each player to the final outcome. In ML, each feature is assigned an importance value representing its contribution to the model's output. The Shapley value is the average marginal contribution of a feature value across all possible combinations in the feature space (47). Feature explainability is of significant importance since it can explain relations of complex features of an ML problem that cannot be seen with the naked eye. In our case, the summary plot was computed on the biomarkers with the best performance in terms of accuracy.

## 3 Results

The performance evaluation metrics for the prediction of IDH mutation are summarized for radiomics features obtained with the Gamma fitting method and with the leakage correction algorithms in Tables 2, 3 respectively.

**Table 2:** Classification metrics ± standard deviation per parametric map from the gamma fitting algorithm.

**Parametric map**	**Sensitivity**	**Specificity**	**F1_score**	**ACC**	**AUROC**
rCBF	46.1 ± 10.7	69.8 ± 22.8	41.7 ± 11.7	63.7 ± 17.8	62.9 ± 15.5
rCBV	68.6 ± 11.3	58.1 ± 18.9	48.8 ± 12.3	60.6 ± 15.6	62.8 ± 13.1
rMTT	31.3 ± 11.3	80.5 ± 12.4	33.2 ± 9.4	68.1 ± 8.0	55.9 ± 8.2
MSI	51.3 ± 16.8	82.4 ± 12.9	50.0 ± 10.5	74.3 ± 7.7	74.2 ± 7.4
TMAX	41.3 ± 20.0	78.1 ± 11.5	37.8 ± 11.7	68.7 ± 5.2	61.2 ± 6.6

**Table 3:** Classification metrics ± standard deviation per parametric map from the leakage correction algorithm.

**Parametric map**	**Sensitivity**	**Specificity**	**F1_score**	**ACC**	**AUROC**
nrCBV	52.5 ± 28.9	62.2 ± 15	37.8 ± 21.1	59.3 ± 11.5	61.5 ± 15.3
K1_unidir_	21.6 ± 13.7	85.7 ± 9.3	24.7 ± 15.4	69.3 ± 5.7	59.2 ± 7.76
K2_unidir_	56.1 ± 5.5	74.7 ± 5.2	48.9 ± 2.7	70 ± 3.1	71.7 ± 5.89
nrCBV_unidir_	59.1 ± 25	54.7 ± 20.1	39.1 ± 5.5	55.6 ± 9.3	65.0 ± 5.30
K_1bidir_	38.9 ± 17.8	87.4 ± 7.8	42.8 ± 16.7	75 ± 6.5	70.5 ± 7.93
K_2bidir_	34.4 ± 33.1	74.8 ± 17	27.9 ± 13.8	64.3 ± 4.6	63.9 ± 17.4
K_epbidir_	44.1 ± 13.3	51.2 ± 12.4	30.5 ± 7.0	49.3 ± 8.2	50.7 ± 10.5
nrCBV_bidir_	58.8 ± 17.3	54.9 ± 27.7	41.5 ± 7.9	55.6 ± 16.9	57.9 ± 15.8

**Figure 3:**
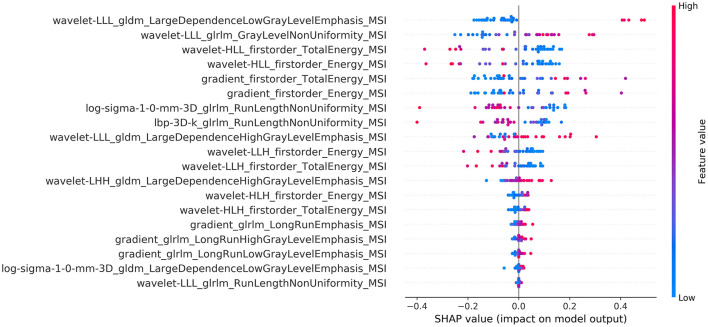
Summary plot of the SHAP values for the best of MSI radiomic features.

**Figure 4:**
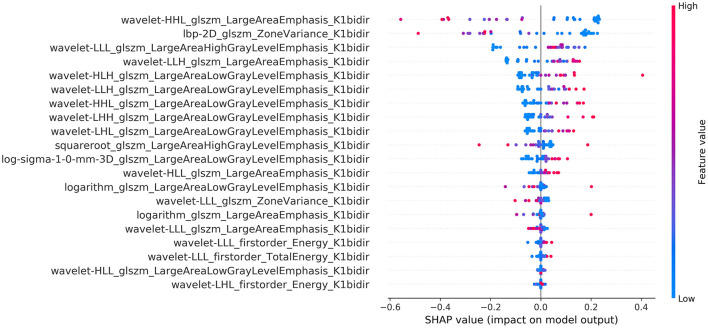
Summary plot of the SHAP values for the best of K1bidir radiomic features.

The calculated summary plot for the features stemming from the MSI and the K1bidir are presented in Figures 3, 4 respectively. More specifically, after feature selection the most prevalent radiomic features are depicted as pseudocolored dots from blue to red. Non-significant features are near a SHAP value of zero. While the distance from zero increases, a higher influence of a specific feature in the prediction performance is denoted meaning that decreased or increased values favor the negative (IDH-mutant) or positive (IDH-wildtype) classes, respectively.

## 4 Discussion

In this work, DSC perfusion qualitative metrics, stemming from different mathematical modeling techniques, were used to quantify the perfusion signal into meaningful imaging markers with the goal to develop a machine learning model for the non-invasive phenotyping of the IDH mutation status. To achieve this, we used an in-house software to calculate the DSC qualitative metrics in order to avoid the variability in the computation of parametric maps, since different software packages can produce results with significant variability in multi-center studies (48–50). In addition, our workflow does not require data harmonization, which can be challenging due to the presence of confounding variables and unknown factors that cause cross-site variations (28).

Driven from the results of Tables 2, 3, the radiomics analysis showed promising results on identifying the IDH mutation status. More specifically, the best candidates that can predict IDH mutation status were MSI with an ACC of 74.3% and an AUCROC of 74.2% and K_1bidir_ with an ACC of 75% and an AUCROC of 70.5%. In addition, most biomarkers showed high specificity while the CBV-related (model dependent) parameters showed high sensitivity. This can be attributed to the imbalanced nature of the dataset, calling for further future research with additional data.

It is notable from the summary plots in Figures 3, 4 that the most important texture features in terms of predictive importance are derived from wavelet analysis for both MSI and K1bidir parametric maps. This clearly indicates that specific frequency bands offer more enhanced discriminatory power compared to the original images. This observation is in line with in other published works concerning IDH classification in gliomas which report high contribution of several wavelet-derived features (21, 51) justifying the need for more research on the effect of frequency filtering in MR images for optimizing IDH mutation prediction. Clinically, detecting biomarkers and tumor subtypes is crucial and a first step in care for patients with gliomas. Biopsies currently serve as the standardized approach for the identification of molecular markers, however due to its invasiveness is not without risk in addition to be subject to sampling errors (52).

Specifically, the identification of IDH mutations is recommended for the classification of gliomas in accordance with the World Health Organization grading system (53). The use of imaging could mean less invasive procedures for grading and surveillance of gliomas (54, 55). Some research has previously suggested that IDH markers can be identified through imaging (56), with DSC multiparametric maps being able to identify angiogenesis transcriptome signatures and pathobiological tumor vasculature which are found in previously identified IDH mutant gliomas (10, 11). Thus, as the IDH gene mutation can reflect changes in metabolism, cellularity or angiogenesis and if this is identifiable with non-invasive imaging, which this study has shown (57); this could result in more accurate presurgical diagnoses and patient management. The latter may improve treatment planning from the initial presentation and treatment monitoring in the clinical and in drug trials settings (58). For example, the knowledge that a tumor is a glioma with an IDH mutation may favor a more aggressive surgical resection, as recent studies suggest that a larger area of resection independently correlates with survival rates in IDH-mutant astrocytic gliomas (59). In addition, accurately predicting IDH mutations may be useful for predictive factors associated with treatment response, as IDH mutated gliomas have shown to have a better response to current standadised treatment (Temozolomide) than non-IDH mutant gliomas (60, 61). According to a recent review on radiomics analysis predicting IDH, an accuracy of 83% was presented when multicentric and multiparametric MRI were incorporated in the study in low grade gliomas (62). Also, as described in the introduction, only two studies include solely DSC for the prediction of IDH. The first work from Sudre et al. (18) reported an ACC of 71%. The second work by Manikis et al. (21) reported an ACC of 70.6% after the normalization of the DSC sequence data in two timepoints. Considering these well documented constrains of the latter work, the pre-processing steps toward IDH classification, this study focused on applying radiomics analysis directly on the parametric maps with the same dataset as of Manikis et al. (21). This effort, led to an increased accuracy up to 5% compared with (21) discarding the need for data harmonization since the radiomic analysis is applied to the parametric maps that have been produced with the same algorithm. This result was achieved by the SVM classifier. The classifiers that have been introduced for the same problem by the abovementioned works incorporate classifiers such as Logistic Regression, Adaptive Boosting, K-Nearest Neighborhood and Random Forest.

A whole brain scan was achieved in acquisition time below 2 seconds while adjusting the rest of the parameters for optimal spatial resolution given the software and hardware constraints of each scanner. Taking that into account, the derived parametric maps are independent from imaging parameters This is an important contribution of this work since it is well-known that in multi-centric studies imaging data from different vendors and protocols introduce significant variability in MRI image quality, contrast and intensity range that affects radiomics analyses. While there are remedies for this problem, there is still no standardized harmonization pipeline hampering trustworthiness and clinical adoption in multisite studies (63, 64). The proposed analysis is based on producing physiology-driven parametric maps from DSC MRI before radiomics extraction, which can be easily standardized across centers if the same software is used.

The major limitation of this study is the high imbalance in IDH mutation status (41 IDH-mutant and 119 IDH-wildtype cases) which occurs due to the natural prevalence of the disease. Furthermore, another limitation of our work is that our radiomic model does not work with non-enhancing-anaplastic gliomas since perfusion curves are absent and cannot produce parametric maps. In future radiomics studies, it could be interesting to investigate a meta-model which combines the highly specific outputs such as K1 with the highly sensitive parameters such as nrCBVunidir and nrCBVbidir and rCBV from gamma fitting algorithm for an overall more robust model. However, a crucial step for future advancement of imaging biomarkers will be the correct and consistent use of internationally standardized and accepted quality criteria, terminology and definitions within the field of advanced neuroimaging and radiomics. In this study the AIF selection was performed manually by the experts from the anterior cerebral artery as the mean value of all voxels inside the AIF ROI. In a future relevant work we could include an automated AIF selection software to possibly achieve more stable fitting performance and therefore more accurate parametric maps avoiding user dependency (65). In addition, it could be interesting to see the IDH classification performance on radiomics features obtained from parametric maps stemming from model-independent deconvolution techniques. If the intra-tumoral perfusion curve differs from the model used, there will be large errors in the perfusion estimate. This will enable a more robust and reproducible signal analysis scheme for facilitating clinical translation.

In conclusion, we developed a fully automated procedure for the characterization of the IDH mutation status from the DSC imaging markers. Despite the variability of the multi-centric data, the analysis was focused directly on the imaging biomarkers, without the use of complex histogram oriented or other normalization techniques. This framework has the potential of becoming an objective diagnostic support tool exploiting the mathematical modeling of the DSC signal to characterize IDH mutation status, which can aid in the diagnosis and management of gliomas.

## Data availability statement

The data analyzed in this study is subject to the following licenses/restrictions: some of the data might contain personal information. Of course we can share the DSC data upon a reasonable request. Requests to access these datasets should be directed to GI, geo3721@ics.forth.gr or grs.ioannidis@gmail.com.

## Ethics statement

The studies involving humans were approved by University College London/University College London Hospitals Joint Research Office (reference number: 213920) and the assigned North West–Liverpool Central Research Ethics Committee (reference number: 18/NW/0395). The studies were conducted in accordance with the local legislation and institutional requirements. The participants provided their written informed consent to participate in this study.

## Author contributions

GI: study concept and design, mathematical modeling of the DSC curves, ML classification, interpretation, and writing of the draft. LP: literature review, writing the clinical part of the draft, and final revision. MI, KS-P, and MW: full text review, visually inspection of the produced tumor ROIs, and final revision. SB and KM: supervision and writing—review and editing. All authors contributed to the article and approved the submitted version.
